# Supplementary material for: Conformation selection by ATP-competitive inhibitors and allosteric communication in ERK2
Source: eLife. 2024 Mar 27;12:RP91507. doi: 10.7554/eLife.91507 (PMC10972564; doi:10.7554/eLife.91507)
Supplement: Supplementary file 3. [file elife-91507-supp3.docx]

**X-ray data collection and refinement statistics**

|  | 2P-ERK2:Inhibitor #8 | 2P-ERK2:Inhibitor #16 |
| --- | --- | --- |
| PDB code | 8U8K | 8U8J |
| Data Collection: |  |  |
| Space group | P2_1_2_1_2_1_ | P2_1_2_1_2_1_ |
| Unit cell parameters (Å,°) | a = 41.98, b = 77.28,  c = 151.54, β = 90.0 | a = 42.10, b = 76.66,  c = 151.97, β = 90.0 |
| Resolution (Å) | 21.30 – 2.10  2.17 – 2.10 | 19.56 – 2.10  2.21 – 2.10 |
| Unique reflections (last shell) | 29,455 | 25,560 |
| Data Completeness (%) | 99.5 (21.30 – 2.10) | 86.5 (19.56 – 2.10) |
| Data Redundancy | 6.50 | 5.66 |
| R_sym_ | 0.15 | 0.15 |
| < I/σ(I) > | 4.60 (at 2.09 Å) | 3.66 (at 2.09 Å) |
| Refinement program | PHENIX 1.20.1_4487 | PHENIX 1.20.1_4487 |
| R_work_, R_free_ | 0.174 , 0.213 | 0.177 , 0.227 |
| R_free_ test set | 1497 reflections (5.08%) | 1299 reflections (5.08%) |
| Wilson B-factor (Å^2^) | 25.9 | 27.4 |
| Anisotropy | 0.116 | 0.125 |
| F_o_, F_c_ correlation | 0.95 | 0.95 |
| Total residues | 349 | 352 |
| Total atoms, non-hydrogen | 3,138 | 3,131 |
| Protein atoms, non-hydrogen | 2,858 | 2,873 |
| Ligand heteroatoms | 30 | 27 |
| Water molecules | 250 | 231 |
| Bond length RMSZ  # \|*Z*\| > 5 | 0.37  0 / 2898 | 0.38  0 / 2913 |
| Bond angles RMSZ  # \|*Z*\| > 5 | 0.56  0 / 3924 | 0.57  1 / 3944 |
| Average B, all atoms (Å^2^) | 27.0 | 27.0 |
